# Sex Differences in the Relationship between Personal, Psychological and Biochemical Factors with Blood Pressure in a Healthy Adult Mexican Population: A Cross-Sectional Study

**DOI:** 10.3390/jcm13020378

**Published:** 2024-01-10

**Authors:** Blanca Estela Ríos-González, Ana Míriam Saldaña-Cruz, Sergio Gabriel Gallardo-Moya, Aniel Jessica Leticia Brambila-Tapia

**Affiliations:** 1 Unidad Médico Familiar #92, Instituto Mexicano del Seguro Social (IMSS), Guadalajara 44340, Mexico; blanca2282@hotmail.com; 2 Especialidad en Medicina Familiar, Centro Universitario de Ciencias de la Salud (CUCS), Universidad de Guadalajara, Guadalajara 44340, Mexico; 3 Departamento de Fisiología, Centro Universitario de Ciencias de la Salud (CUCS), Universidad de Guadalajara, Guadalajara 44340, Mexico; 4 Programa de Doctorado en Farmacología, Departamento de Fisiología, Centro Universitario de Ciencias de la Salud (CUCS), Universidad de Guadalajara, Guadalajara 44340, Mexico; sergio.gallardo@alumnos.udg.mx; 5 Departamento de Psicología Básica, Centro Universitario de Ciencias de la Salud (CUCS), Universidad de Guadalajara, Guadalajara 44340, Mexico

**Keywords:** systolic blood pressure, diastolic blood pressure, biochemical variables, psychological factors, anthropometric factors, sex

## Abstract

Hypertension is one of the main risk factors related to cardiovascular mortality, being the levels of blood pressure (BP) related to a variety of personal, anthropometric, biochemical and psychological variables; however, the study evaluating the association of all these factors in systolic blood pressure (SBP) and diastolic blood pressure (DBP) in a sample of relatively healthy subjects has not been performed. The aim of the study was to determine the main variables associated with SBP and DPB in a sample of relatively healthy subjects. A total of 171 participants were included, in which personal, anthropometric, positive and negative psychological variables and biochemical variables were measured. We observed that men showed higher levels of SBP and DBP than women, with more differences for SBP. Among the biochemical factors and SBP, we found that albumin and monocytes were positively correlated with it, while potassium, phosphorus and eosinophils were negatively correlated with it. Additionally, schooling was a constant variable negatively correlated with SBP in all samples (global, men and women). Among psychological variables, we observed that emotional perception was negatively correlated with SBP in men’s and women’s samples, while autonomy was positively correlated with SBP in the men’s sample; however, their association was less when compared with the personal and biochemical variables included in the multivariate model. With regard to DBP, we observed that the biochemical variables, hemoglobin, sodium, uric acid and glucose, were positively correlated with DBP in the global sample, while chloride and BUN were negatively correlated with it. In addition, many personal and behavioral variables, including BMI, age and smoking consumption frequency, also correlated with DBP in the global sample. In conclusion, BP is affected by different factors, and these affect each sex differently.

## 1. Introduction

High levels of blood pressure (BP) are associated with cardiovascular mortality since high levels are associated with a greater probability of acute myocardial infarction or stroke [1]. BP can be modified by several factors, including personal (age and ethnicity), anthropometric (those related to body adiposity), biochemical (blood count cells, uric acid and serum electrolytes) and psychological ones [1,2,3,4,5,6]. In the case of psychological factors, this relationship is explained by considering that emotions affect BP by impacting the sympathetic nervous system and the hypothalamic–pituitary–adrenal axis [2]. Norepinephrine is an indirect marker of sympathetic tone, and this is usually elevated in hypertensive patients, both in the sympathetic nerve terminals as well as in the urine. In addition, high levels of noradrenaline and dopamine have also been observed in depression [7]. This shows a link between emotional states and hypertension. In addition, a previous report showed that variations in depression and mental health in people with hypertension and metabolic syndrome produced a modification in BP over time [8]. Another report showed that emotion recognition ability was higher in normotensive people when compared with pre-hypertensives and hypertensives [9]. Furthermore, a recent report showed that anxiety disorders and depression are associated with resistant hypertension [10]. Finally, it was recently demonstrated that trait anger was associated with increases in BP in an experimental group of hypertensive people [11]. 

On the other hand, it has been shown that serum electrolytes and biochemical parameters influence BP. A study performed in hypertensive and normotensive men showed that hypertensive men had higher levels of glucose, cholesterol and triglycerides, as well as higher levels of sodium, chloride and potassium when compared with normotensive men [12]. Additionally, high salt intake has been related to an increase in BP and arterial stiffness [13], while potassium intake has been associated with BP-lowering effects, mainly in hypertensive people [3]. However, a study searching for the association between personal, biochemical, anthropometric and psychological factors in order to determine their correlation with systolic blood pressure (SBP) and diastolic blood pressure (DBP) in relatively healthy people has not been performed. This study is necessary in order to understand the relationships among all these variables with BP when adjusted with the other ones. 

Therefore, our objective was to determine the association between personal, anthropometric, biochemical and psychological variables, including positive (positive emotions, well-being, optimism and emotional intelligence) and negative (negative emotions, depression and anxiety) psychological factors, with BP in a sample of a relatively healthy population.

The hypotheses were: (1) There are sex differences in BP, with men having higher levels of it; (2) Presence of negative psychological factors are associated with higher BP, while positive factors are associated with lower BP, after adjustment for biochemical factors; (3) The association of psychological, personal, anthropometric and biochemical factors in BP impacts differently between sexes.

## 2. Subjects and Methods

### 2.1. Ethical Considerations

The study was conducted according to the guidelines of the Declaration of Helsinki and was approved by the ethical committee of the Health Sciences University Center, with the registration number: 19–21, approval date: 14 October 2019. All the participants signed an informed consent. 

### 2.2. Subjects

The inclusion criteria were: (a) to be older than 18 years old, (b) do not have chronic or acute diseases known by the subject (self-reported), (c) subjects who were not consuming illegal drugs, (d) subjects who were not consuming hormonal products to increase muscular mass, (e) subjects who were not pregnant, (f) subjects who were not genetically related to another participant of the study (i.e., brothers, cousins), and (g) subjects who preferably did not smoke. The elimination criterium was: the absence of the measurement of any variable. 

#### 2.2.1. Study Design: Cross-Sectional Study

This study consists of the measurement of several variables at the same point in time in order to establish possible relationships between them. 

#### 2.2.2. Procedures

The invitation was performed through the distribution of an announcement via social networks (WhatsApp, Facebook); additionally, in order to complete the minimum sample size, we invited university students personally. All the potential participants met the inclusion criteria; if they accepted to participate, they were cited in computer rooms of the University of Guadalajara, where they signed informed consent and filled out Google Forms questionnaires which included personal, behavioral and psychological variables. After filling out the questionnaire, the anthropometric indexes, body mass index (BMI) and waist-to-hip ratio (WHR), were obtained, along with the SBP and DBP measurements. 

The blood and samples were obtained by trained personnel who worked for a certified laboratory. All the samples were transported to a certified laboratory, where the biochemical analyses were performed. No payment was offered to participants. As an advantage for participating, their biochemical results were sent to them along with a medical interpretation. In this sense, it is important to clarify that all the surveys were filled on computers and in person, where any doubt could be clarified by the research team.

#### 2.2.3. Sample Size 

The sample size was calculated with the formula for bivariate correlations [14], which was estimated to detect a statistical confidence of 95% and a statistical power of 80% for a minimum correlation value of 0.2, which means a very low correlation. This formula yielded a total of 67 subjects. However, the minimum sample size intended was 80 individuals per sex. In this sense, we used this formula in order to detect very low bivariate correlations as significant, and we did not adjust for a specific number of independent variables because no formulas for multivariate analyses were found. However, with this formula, we expected to detect clinically relevant correlations in the bivariate and multivariate analyses. 

When multivariate analyses are carried out, a large sample size is desirable in order to avoid false results, with a minimum of 10 participants per variable; nevertheless, when it is not reachable, multivariate analyses can also be performed in order to diminish the confusion bias produced by the influence of multiple variables in a dependent variable, being cautious with the interpretation.

### 2.3. Personal Variables 

The following personal and behavioral variables were measured: age, sex, schooling, whether they had a job, a romantic partner, and children, socioeconomic level, daily hours of physical activity, daily free hours, and monthly extra money. The monthly extra money variable was measured with 5 categories, ranging from nothing to more than USD 180. The frequency of alcohol and smoking consumption was measured with 5 categories: from never to 4 or more times in a week. Sleep satisfaction was measured with the first item of the OVIEDO sleep questionnaire, with the answer options ranging from 1 (very unsatisfied) to 7 (very satisfied); sleep quality was measured with the second item (which included 5 items) of the OVIEDO sleep questionnaire, which ranged from 1–5 (low sleep quality to high sleep quality) [15]. We measured the quality of food intake with the Mini-Ecca scale, which ranged from 1–12 (very low quality to very high quality) [16]. We also measured two questions of eating behavior: (a) the frequency of food consumption in excess and (b) the frequency of food consumption outside the home. Both questions were measured with 7 answer options, ranging from 1 (less than once in a month) to 7 (all the days); these two questions were obtained from the eating behavioral questionnaire [17]. 

### 2.4. Measurement of Anthropometric Variables 

The height and weight were obtained by trained personnel with a Tanita brand scale (model bc-533) and measuring tape attached to the wall to calculate the BMI. The hips and waist circumferences were also obtained by trained personnel by using a measuring tape. These measurements were used to calculate the waist/hip ratio (WHR). 

### 2.5. Measurement of Blood Pressure 

The measurements of SBP and DBP of the participants were performed with the subject sitting with the left arm placed in semiflexion at the level of the hearth. This was performed through an automated procedure by using an upper arm BP monitor brand Omron (model Hem-7320).

### 2.6. Psychological Variables

We measured the following psychological variables: depressive symptoms, with the 10-items CES-D scale, ranging from 1 (none of the days) to 4 (all the days) [18,19]; anxiety symptoms, with the Generalized Anxiety Disorder test (GAD-7), ranging from 0 (never) to 3 (almost all the days) [20]; the presence of positive and negative emotions, with the positivity-self scale (PSS), ranging from 1 (never) to 5 (almost always) [21]; the 6 subscales of the shortened version of the scale of psychological well-being (PWB) (including self-acceptance, environmental mastery, autonomy, personal growth, purpose in life and positive relations with others), with the scale ranging from 1 (totally disagree) to 6 (totally agree) [22]; optimism was measured with the Life Orientation Test (LOT-R), ranging from 1 (totally disagree) to 5 (totally agree) [23]; additionally, we measured 5–6 items of 4 subscales of the Trait Emotional Intelligence Questionnaire (TEIQUE), which included: emotion perception (5 items), self-motivation (5 items), emotion regulation (6 items) and assertiveness (6 items), which ranged from 1 (totally disagree) to 7 (totally agree). These items are described in the Supplementary file [24]. 

### 2.7. Biochemical Variables Measurement

Analyses of biochemical variables were performed in a certificated laboratory. Blood samples were obtained to quantify the following: (1) complete blood count test (including leucocytes and their subpopulations, erythrocytes and hematocrit), (2) complete lipid profile test (total cholesterol, low-density lipoprotein (LDL), high-density lipoprotein (HDL) and triglycerides), (3) liver function tests (gamma-glutamyl transferase (GGT), aspartate aminotransferase (AST), alkaline phosphatase (ALP), alanine aminotransferase (ALT), lactate dehydrogenase (LDH) enzymes and direct and indirect bilirubin, albumin and total proteins), (4) blood chemistry (creatinine, glucose, urea, uric acid and blood urea nitrogen (BUN)), (5) serum electrolytes (including calcium, phosphorus, magnesium, iron, sodium, potassium and chloride) and (6) pancreatic enzymes (amylase and lipase). All the values detected that were out of range were double-checked in order to verify them.

### 2.8. Statistical Analysis 

In order to perform the description of continuous variables, mean and standard deviations were used when the distribution was parametric, and median and ranges when it was non-parametric. In order to compare sociodemographic variables between the sexes, we used the chi-squared test for categorical variables and the Student’s *t*-test or Mann–Whitney U test for continuous ones (for parametric or non-parametric distribution, respectively). In order to compare categories of SBP and DBP, as well as WHR and BMI, we used chi-squared and Fisher’s exact tests. In order to correlate quantitative variables with SBP and DBP, we used Pearson and Spearman correlation tests, depending on the parametric or non-parametric distribution of the data.

In order to detect the association of independent variables with SBP and DBP, we performed multiple linear regression analysis (using the stepwise method). This analysis was performed by considering that the dependent variables (SBP and DBP) were continuous and normally distributed, and the independent variables were continuous, dichotomic and ordinal. The stepwise method was selected in order to obtain a model with all the included variables being significant. This analysis was performed for all the samples and segmented by sex. The segmented analyses were performed in order to detect specific correlations between independent and dependent variables in each sex. We did not perform interaction analyses between sex and each independent variable in order to better understand the results, analyzing the effect of each variable (without interactions) on the dependent variables in each sex. This decision of doing stratified analyses by sex was performed a priori.

The determination of multicollinearity was performed in order to avoid collinear variables in each multivariate model, and this was confirmed by including only variables with tolerance values above 0.3. 

In addition, we performed the Cronbach’s alpha test for all the psychological instruments in order to obtain the reliability of each scale and subscale used. All analyses were performed with the software SPSS v.25, and a *p*-value < 0.05 was considered significant. 

## 3. Results

A total of 171 participants were included, from whom 91 (53.2%) were women; the mean ± SD of the age of the sample was 27.17 ± 10.68. All scales and subscales employed had a Cronbach’s alpha test >0.6.

The descriptive data of sociodemographic and psychological variables are described in Table 1, where we observe that all sociodemographic variables were similar between the sexes. We observed some differences in psychological variables between the sexes, where anxiety and negative emotions were significantly higher in women than in men, and assertiveness and autonomy were significantly higher in men than in women (Table 1). In Table 2, we show the descriptive data of SBP and DPB, where we could detect significantly higher levels of both variables in men when compared with women. These differences were corroborated when the values were categorized as normal, pre-hypertension or hypertension, according to the values proposed by the Joint National Committee 7 (JCN-7) [25]. The highest differences between the sexes were observed for SBP, where men showed 10 times more frequency of individuals in the pre-hypertension category when compared with women (Table 2). We also showed the descriptive data of BMI and HWR, where we found a significant difference between the sexes, with higher levels of WHR in men than in women. This was compared with the numeric values and the values categorized as normal or high, according to the desirable WHR values for the Mexican population [26]. 

The descriptive data of biochemical variables are described in Table 3, and the values out of the reference range are also mentioned. The laboratory ranges are according to the desirable values and not the normal values found in the general population; therefore, we included the values out of the reference range in the analyses. In addition, all the values out of range detected in the laboratory tests were double-checked (performed twice) in order to verify them. 

### 3.1. Bivariate Correlations 

In the bivariate correlations between the studied variables and SBP/DBP performed in the whole sample (Table 4), we observed that the variable most positively correlated with SBP was male sex (r = 0.607), followed by uric acid (r = 0.522), WHR (r = 0.482) and creatinine levels (r = 0.459); we also observed that all liver enzymes showed low but positive significant correlations with SBP, with the highest correlation value for GGT (r = 0.369). Electrolyte levels also showed significant correlations with SBP, with a low positive correlation between SBP and calcium levels (r = 0.222) and low negative correlations with phosphorus and chloride levels (r = −0.246 and r = −0.217). The psychological variable “autonomy” showed a low positive correlation with SBP (r = 0.221), while “depression” showed a low negative correlation with it (r = −0.164). 

In the case of DBP, we observed that the most associated variable was uric acid (r = 0.347), followed by hemoglobin (r = 0.346), erythrocytes (r = 0.325), hematocrit (r = 0.336), WHR (r = 0.341), male sex (r = 0.322), GGT (r = 0.326) and total proteins (r = 0.250). The electrolytes calcium, sodium and magnesium showed low positive correlations with DBP, while phosphorus showed a very low negative correlation with DBP (Table 4). The only psychological variable associated was “autonomy”, with a very low positive correlation with DBP (r = 0.162). 

### 3.2. Multivariate Regression Analysis for SBP 

In the multivariate regression analysis for SBP in the whole sample (Table 5), we observed that male sex was the most positively associated variable with SBP, followed by BMI and age, while schooling was negatively correlated with SBP. In the case of biochemical variables, we observed that albumin, monocytes, glucose and creatinine were positively correlated with SBP, while phosphorus, potassium and eosinophils were negatively correlated with it, with a robust R of the model: 0.770. 

In the multivariate regression analysis for SBP performed in women (Table 6), we observed that BMI and age were the most positively associated variables with SBP, while schooling, eosinophils, ALP, emotion perception and having children were variables negatively correlated with SBP, with an R of the model of 0.673. 

Finally, in the multivariate regression analysis for SBP in men (Table 7), we observed that total proteins, daily physical activity hours, autonomy, monthly free money and calcium were positively correlated with SBP, while schooling, phosphorus, potassium, iron, frequency of alcohol consumption, socioeconomic level and emotion perception were negatively correlated with it. The R of the model was also robust at 0.757.

### 3.3. Multivariate Regression Analysis for DBP

In the multivariate regression analysis for DBP in the global sample, we detected that the positively correlated variables were age, hemoglobin, glucose, LDH, BMI, sodium and magnesium, while the negatively correlated variables with DBP were BUN, schooling, chloride and frequency of smoking consumption. The R of the model was 0.644 (Table 8). 

In the multivariate analysis for women, we observed many associated variables (Table 9) that formed a robust model (R = 0.768), with the following positive associated variables with DBP: uric acid, LDH, hemoglobin, age, daily free hours, sleep satisfaction and BMI; and the following negatively correlated variables with DBP: phosphorus, with romantic partner, daily physical activity hours, personal growth, triglycerides, WHR, chloride, with children and frequency of smoking consumption. 

In the multivariate analysis for DBP in men, the positively correlated variables with DBP were with children, total proteins, magnesium, self-motivation and AST, while the negatively correlated variable with DBP was quality of food intake. The R of the model was 0.591 (Table 10).

## 4. Discussion 

### 4.1. Sex Differences in Blood Pressure 

In the present study, we observed that most personal (including BMI) variables were similar between the sexes, which makes both groups comparable in BP parameters. We showed that both SBP and DBP were significantly higher in men and were correlated by different factors in both sexes. The higher values of SBP and DBP in men coincide with previous reports showing higher levels of pre-hypertension (for SBP) in men when compared with women in the adult population of China (71.1% vs. 44.6%) [27] and the university population of Spain (56.5% vs. 13.0%) [1]. These results coincide with ours, where higher levels of hypertension (11.2% vs. 2.2%) and mainly pre-hypertension of SBP (47.5% vs. 4.4%) were found in men when compared with women. These results are more similar to that found by Ortiz-Galeano et al. in Spain [1]. This is explained by the higher similitude between our study and their study, being both populations mainly comprised of young people, where sex differences are more pronounced. The differences in BP observed between the sexes have been explained by the biological effects of sex chromosomes, including sex hormones and reproductive events [4]. However, there is evidence that cardiovascular complications start at lower BP levels in females than in males [4], questioning the current practice of using the same BP threshold for the identification of hypertension in both sexes [28,29]. In addition, in the elderly, hypertensive women double the number of men [30]; this can be partly explained by hormonal and hemodynamic changes that occur after menopause, including a higher sympathetic activity and vasoconstrictor responsiveness [31]. 

### 4.2. Personal and Biochemical Factors in SBP

In the bivariate correlations performed in the whole sample, we observed that male sex was the variable most associated with SBP (Table 4), which coincides with the sex differences of SBP previously mentioned. In addition, we observed that many biochemical and some psychological variables were positively and negatively correlated with SBP and DBP. 

In the case of SBP, in the multivariate regression analysis for the global sample, we observed that male sex, BMI and age were the most positively correlated variables with SBP, followed by schooling, which was negatively correlated with SBP. BMI and age are well-known variables related to high SBP, which, in the case of BMI, is explained by the role that adiposity plays in the physiopathology of hypertension [5]. This variable, along with age, was positively correlated with SBP in the whole sample, as well as in the women’s sample (Table 5 and Table 6), which suggests that their association, although present in both sexes, is higher in women than in men. In the case of the negative correlation of schooling with SBP, a possible explanation is that people with higher schooling remain sited or physically inactive more often, which negatively impacts SBP (when adjusted for BMI and the rest of the searched variables). This variable was also seen in men’s and women’s subsamples (Table 6 and Table 7) and coincides with the positive correlation of the variable daily physical activity hours with SBP in the men’s sample. These findings coincide with a previous report performed in normotensive men, where significant correlations between SBP, physical activity and left ventricle mass were found [32]. Different to that found in hypertensive men in whom physical activity is associated with reduced BP [33], the coincidence between these findings and our results is explained by the fact that most men included in this study were normotensive or pre-hypertensive, and only 8.8% were hypertensive. These findings indicate the importance of personal and behavioral variables in BP variations of a relatively healthy population. We also observed that the personal variable “sleep satisfaction” was marginally and positively correlated with SBP in the whole sample (Table 5), which suggests that restful sleep is needed in order to maintain a healthy BP and avoid hypotension. In addition, it has been shown that poor sleep patterns are related to a higher probability of presenting hypertension [34]. Therefore, it seems that sleep quality has an important role in BP regulation. 

We also observed that the biochemical variables albumin and total proteins were positively correlated with SBP in the multiple regression analyses of global and men’s samples (Table 5 and Table 7), which is probably due to the oncotic pressure that albumin exerts [35]. Additionally, these results coincide with the bivariate correlations between total proteins and albumin with SBP in the whole sample (Table 4). On the other hand, the serum electrolytes were also correlated with SBP in the multivariate regression analyses, where phosphorus and potassium were negatively correlated with it in the global and men’s samples (Table 5 and Table 7). These findings coincide with previous reports, showing that phosphorus and potassium supplementation are related to lower levels of SPB [3,6]. In the men’s sample, we also observed that iron was negatively correlated with SBP, while calcium was marginally positively correlated with SBP (Table 7). The negative correlation between iron and SBP in men coincides with a report showing that intake of non-heme iron is inversely related to BP [36]. It is interesting that this correlation was observed only in men and opposed the bivariate correlation between SBP and iron in the global sample, which suggests that men are mainly affected by the influence of iron in SBP, although additional studies should corroborate it. In addition, the marginal positive correlation between calcium and SBP in men, which, although coincides with the bivariate correlations in the global sample, contrasts with this last study that found that calcium intake was also inversely correlated with BP [36] and with a report showing a low negative correlation between calcium in serum with SBP and DBP in patients with type 2 diabetes [37]. However, a previous report performed in Pakistan showed that people with essential hypertension had higher concentrations of many electrolytes, including calcium when compared with normotensives [38]. Therefore, more studies searching the relationship between calcium concentration and BP are needed. 

We also observed that the global and women’s samples showed that monocytes were positively correlated with SBP, while eosinophils were negatively correlated with it (Table 5 and Table 6). These findings coincide with a previous report showing a positive association of monocytes and neutrophils with higher levels of SBP and eosinophils with lower levels of SBP [39]. This is an interesting finding that needs to be further explored and which suggests a role of inflammatory mechanisms in SBP levels. Finally, a positive correlation between creatinine and SBP in the whole sample was observed (Table 5), which coincides with the positive significant bivariate correlations between creatinine and SBP and DBP in the global sample (Table 4) and suggests that renal function plays an important role on SBP. 

### 4.3. Psychological Factors in SBP 

With respect to the psychological variables studied, we found that there were no psychological factors associated with SBP in the global sample; however, in the sex-specific analysis, the psychological variable “emotion perception” was negatively associated with SBP in both sexes in the multivariate analyses (Table 6 and Table 7); in addition, in the men’s sample, there was a positive correlation between the psychological variable “autonomy” with SBP in the multivariate analysis (Table 7). The negative correlation with “emotion perception” coincides with a previous report showing a higher ability of emotion recognition in normotensive subjects when compared with pre-hypertensive and hypertensive subjects [9] and with a study that showed that anxiety disorders and depression were associated with resistant hypertension [10], considering that emotion perception was inversely related to symptoms of anxiety and depression, with moderate negative correlations. However, it is of interest that we did not find a significant correlation between anxiety and depression variables with SBP or DBP in the multivariate analyses. This suggests that these negative psychological variables can affect SBP only when they are present as clinical disorders and not when they are measured with symptomatic scales, as in this case. In addition, these variables have been associated with the presence of hypertension and not with BP variations in a relatively healthy population. 

The positive correlation between the psychological variable “autonomy” with SBP in the men’s sample could be related to the fact that this variable is related to being less influenced by other’s opinions in one own’s life, as well as with a higher ability to defend one own’s rights. Therefore, men with higher levels of this ability can be prone to having higher levels of SBP because they have a “stronger” character and willpower; however, further studies evaluating the influence of this variable on SBP in each sex are needed. 

### 4.4. Personal and Biochemical Factors in DBP

The results of DBP showed that some similar and many different personal and biochemical variables were significantly associated with it. As observed in SBP, BMI and age were positively correlated with DBP in the global and women’s samples (Table 8 and Table 9), suggesting that these two variables (age and BMI) influence women more than men.

The positive correlations of hemoglobin, sodium and magnesium and the negative correlations of chloride with DBP are of interest. In the case of hemoglobin, the results coincide with a report showing that higher hemoglobin levels are related to an increase in SBP and DBP in both sexes [40]. Data coincide with the bivariate analyses in this study, where moderate significant correlations were found between hemoglobin and SBP and DBP in the global sample (Table 4), and also coincide with positive correlations between SBP and DBP with erythrocytes and hematocrit. This correlation has been explained by the effects of hemoglobin in the increase in blood viscosity, which, in turn, is related to increased peripheral resistance and BP; in addition, higher hemoglobin levels have been related to less secretion of B-type natriuretic peptide, which is related to natriuresis and aldosterone inhibition, leading to reduced BP. Therefore, by an opposite mechanism, an increase in hemoglobin levels would increase BP [40]. It is interesting that hemoglobin appeared in the multivariate analyses of DBP but not of SBP, for which the bivariate correlation with hemoglobin is higher (Table 4). It is possible that with larger sample sizes, hemoglobin could also be correlated with SBP, as in the previous report [40]. With respect to the negative correlation between chloride and DBP in the global sample, we observed that these results coincide with a previous report showing that serum chloride levels were inversely correlated with SBP and DBP [41]. The positive correlation with sodium coincides with a report showing that high sodium intake is related to an increase in BP [13] and with a report that demonstrated higher levels of sodium in hypertensive people [12]; in addition, the marginally positive correlation between magnesium and DBP coincides with the low but significant positive correlation between magnesium levels and DBP in the global sample of the bivariate analysis (Table 4), although differing from a study showing that magnesium intake was inversely related with BP [36], and with another study showing that magnesium supplementation is related to BP reduction in patients with mild hypertension [42]; however, a study performed in persons with essential hypertension showed higher levels of magnesium in this population when compared with normotensive persons [38]. These discrepancies can be explained by the type of population studied in each report, suggesting that serum magnesium could be positively correlated with DBP in a relatively healthy population. However, only larger studies will clarify this relationship. Finally, in the multivariate analysis for DBP in the whole sample, we found that BUN was negatively correlated with it in a significant way. The study of this relationship was not found in previous reports; therefore, future research will clarify the role of BUN in DBP. 

When we observed the variables associated with DBP in each sex, uric acid was the most associated variable with DBP in women (Table 9), which coincides with a previous report showing that serum uric acid levels were only associated with BP in women [43]. In addition, as previously reported [12,43], we observed a positive correlation between fasting glucose with SBP and DBP in the global samples (Table 5 and Table 8) and with SBP in the women’s sample (Table 6), indicating that the association between glucose and BP is higher in women than in men, as previously suggested [43]. 

The positive correlation between LDH and DPB in the multivariate analysis of women, as well as the positive correlation between AST and DBP in the men’s sample, suggest that liver function and its specific enzymes play a role in DBP; however, more studies are needed in order to determine their influence, considering that no related reports were found. Similarly, the negative correlation between triglycerides and DBP and the marginal negative correlation between WHR with DBP in the women’s sample require further research that discards or corroborates these findings. 

### 4.5. Behavioral and Psychological Factors in DBP 

Interestingly, the frequency of smoking consumption was negatively correlated with DBP after adjusting for confounders, and this coincides with a cross-sectional study performed in men that showed that current smokers have lower DBP when compared with nonsmokers [44]. With respect to other sociodemographic and behavioral variables, we observed that different variables were associated with each sex; for example, in women, the variables of having a romantic partner, having children and daily physical activity hours were negatively correlated with DBP, while daily free hours was positively correlated with it (Table 9). The variable having children was positively correlated with DBP in men, in whom the quality of food intake was negatively correlated with DPB (Table 10). These results suggest that personal variables influence DBP more than SBP and should be considered in studies researching variables associated with DBP.

Although no psychological variables were associated with DBP in the multivariate analysis of the global sample, the subscale “personal growth” of the scale “psychological well-being” was negatively correlated with DBP in women, and the subscale “self-motivation” of the TEIQUE scale of emotional intelligence was marginally and positively correlated with DBP in men. The variable personal growth is related to questions like “I have the feeling that over time, I have developed a lot as a person”, and its relationship with lower DPB in women can be related to a more calmed mood and higher mental health. In addition, the variable self-motivation is related to questions like: “On the whole, I’m a highly motivated person”, which could contribute positively to levels of BP and, in this case, DBP. Nevertheless, further studies will clarify this relationship. 

The main limitation of the study is the sample size, which, if larger, would have permitted us to perform analyses separated by normotensives, pre-hypertensives and hypertensives in each sex and would have diminished the possible bias by including many independent variables in the multivariate analyses; in addition, the non-random sampling method did not permit us to perform a generalization to all in the Mexican population, and neither to the population of all ages, considering that most participants were young people. The usage of electronic questionnaires can also diminish the accurate understanding of the questions, which could have affected the answers of the participants. Another limitation is the cross-sectional nature of the study, which cannot permit us to determine causal relations between the variables studied and BP. However, the main strength is the inclusion of many independent variables, including personal, biochemical, anthropometric, behavioral and psychological variables, which led us to detect the influence of each one of these factors in a more accurate way. 

In conclusion, we observed that men showed higher levels of SBP and DBP than women, with more differences for SBP. In addition, we reported that many personal and psychological variables were associated with these variables, with some differences between the sexes. Among the personal variables, BMI and age were significantly and positively correlated with SBP and DBP, with more correlation in the women’s sample. Among the biochemical factors and SBP, we found that albumin and monocytes were positively correlated with it, while potassium, phosphorus and eosinophils were negatively correlated with it. Additionally, schooling was a constant variable negatively correlated with SBP in all samples (global, men and women). Among the psychological variables, we observed that emotional perception was negatively correlated with SBP in men’s and women’s samples, while autonomy was positively correlated with SBP in the men’s sample; however, the association was less when compared with personal and biochemical variables. With regard to DBP, we observed that the biochemical variables, hemoglobin, sodium, uric acid and glucose, were positively correlated with DBP in the global sample, while chloride and BUN were negatively correlated with it. In addition, many personal and behavioral variables, including BMI, age and smoking consumption frequency, also correlated with DBP in the global sample. In addition, many other personal variables were differently correlated with DBP in each sex. All these results indicate that BP is a variable that presents multiple correlations with different factors, including the sex, and these correlations in each sex are different; therefore, studies aimed at identifying or studying BP should consider the effect of sex. Further longitudinal studies with larger sample sizes will corroborate or discard these results.

## Figures and Tables

**Table 1:** Descriptive data of sociodemographic and psychological variables and their comparison between the sexes.

Variable	Women (n = 91)	Men (n = 80)	*p*-Value
Age	27.91 ± 10.99	26.33 ± 10.91	0.334
With romantic partner, n (%)	45 (49.5)	43 (53.8)	0.646
With children, n (%)	24 (26.4)	11 (13.8)	0.057
With job, n (%)	51 (56.0)	38 (47.5)	0.286
Schooling, n (%)			0.582
- Elementary school	1 (1.1)	0 (0.0)	
- Secondary	3 (3.3)	2 (2.5)	
- Preparatory	48 (52.7)	50 (62.5)	
- University (Bachelor’s degree)	32 (35.2)	21 (26.3)	
- Master’s degree	7 (7.7)	6 (7.5)	
- Ph.D. degree	0 (0.0)	1 (1.2)	
Socioeconomic level, n (%)			0.185
- Very low	0 (0.0)	2 (2.5)	
- Low	16 (17.6)	15 (18.7)	
- Average	74 (81.3)	59 (73.8)	
- High	1 (1.1)	4 (5.0)	
- Very high	0 (0.0)	0 (0.0)	
Monthly extra money, n (%)			0.146
- Nothing	6 (6.5)	9 (11.2)	
- Less than USD 60	33 (36.3)	16 (20.0)	
- From USD 61 to USD 120	28 (30.8)	25 (31.3)	
- From USD 121 to USD 180	9 (9.9)	10 (12.5)	
- More than USD 180	15 (16.5)	20 (25.0)	
Smoking frequency, n (%)			0.371
- Never	83 (91.2)	69 (86.2)	
- Two to four times in a year	4 (4.4)	7 (8.8)	
- Once a month or less	1 (1.1)	1 (1.2)	
- Two to three times in a week	2 (2.2)	0 (0.0)	
- Four or more times in a week	1 (1.1)	3 (3.8)	
Alcohol consumption frequency, n (%)			0.612
- Never	14 (15.4)	16 (20.0)	
- Two to four times in a year	28 (30.8)	22 (27.5)	
- Once a month or less	42 (46.2)	33 (41.2)	
- Two to three times in a week	6 (6.5)	9 (11.3)	
- Four or more times in a week	1 (1.1)	0 (0.0)	
Daily free hours, median (range)	4 (0–11)	4 (0–14)	0.012 *
Daily physical activity hours, median (range)	1 (0–5)	1 (0–4)	0.399
Sleep satisfaction (OVIEDO scale), mean ± SD			0.385
- Very unsatisfied	7 (7.7)	2 (2.5)	
- Quite unsatisfied	9 (9.9)	9 (11.3)	
- Unsatisfied	14 (15.4)	15 (18.8)	
- Medium	32 (35.1)	25 (31.2)	
- Satisfied	18 (19.8)	12 (15.0)	
- Quite satisfied	8 (8.8)	9 (11.2)	
- Very Satisfied	3 (3.3)	8 (10.0)	
Sleep quality (OVIEDO scale), mean ± SD	3.55 ± 0.96	3.74 ± 0.93	0.185
Frequency of food consumption outside the home, n (%)			0.024 *
- Less than once a month	8 (8.8)	1 (1.2)	
- Once a month	4 (4.4)	1 (1.2)	
- Once every 15 days	12 (13.2)	11 (13.8)	
- 1–2 times a week	43 (47.3)	29 (36.2)	
- 3–4 times a week	14 (15.4)	15 (18.8)	
- 5–6 times a week	6 (6.5)	13 (16.3)	
- Daily	4 (4.4)	10 (12.5)	
Frequency of food consumption in excess, mean, n (%)			0.115
- Less than once a month	14 (15.4)	5 (6.3)	
- Once a month	10 (11.0)	11 (13.8)	
- Once every 15 days	13 (14.3)	17 (21.3)	
- 1–2 times a week	36 (39.6)	29 (36.3)	
- 3–4 times a week	8 (8.8)	14 (17.5)	
- 5–6 times a week	6 (6.5)	1 (1.3)	
- Daily	4 (4.4)	3 (3.5)	
Quality of food intake (Mini-Ecca scale), mean ± SD	7.70 ± 2.65	7.19 ± 2.39	0.122
**Psychological variables**			
Anxiety (GAD-7), mean ± SD	1.19 ± 0.74	0.90 ± 0.60	0.019 *
Depression (CES-D), mean ± SD	1.96 ± 0.58	1.78 ± 0.45	0.095
Psychological well-being (PWB), mean ± SD			
- Self-acceptance	4.59 ± 1.23	4.83 ± 1.04	0.237
- Autonomy	3.88 ± 0.99	4.35 ± 0.98	0.002 *
- Purpose in life	4.53 ± 1.22	4.65 ± 1.13	0.568
- Positive relations with others	4.88 ± 1.04	4.72 ± 0.99	0.242
- Personal growth	5.08 ± 0.97	5.01 ± 0.92	0.333
- Environmental mastery	4.37 ± 1.10	4.47 ± 0.98	0.524
Emotional intelligence (TIEQUE), mean ± SD			
- Assertiveness	4.63 ± 0.96	5.13 ± 1.10	0.002 *
- Emotion regulation	4.84 ± 1.17	5.04 ± 1.26	0.290
- Self-motivation	5.16 ± 1.19	4.97 ± 1.20	0.300
- Emotion perception	4.94 ± 1.45	5.06 ± 1.38	0.663
Positive emotions (PSS), mean ± SD	3.65 ± 0.65	3.83 ± 0.52	0.051
Negative emotions (PSS), mean ± SD	2.61 ± 0.65	2.39 ± 0.57	0.018 *
Optimism (LOT-R), mean ± SD	3.66 ± 0.74	3.70 ± 0.67	0.848

* *p*-value obtained with chi-squared test, Student’s *t*-test and Mann-Whitney U test. Monthly extra money: five categories, from nothing to more than USD 180. Smoking and alcohol consumption frequencies were measured, from 0–4 (never to more than four times in a week); sleep satisfaction (OVIEDO scale), from 1–7 (very unsatisfied to very satisfied); sleep quality (OVIEDO scale), from 1–5 (low quality to high quality); quality of food intake (Mini-Ecca scale) from 1–12 (very low quality to very high quality); frequency of food consumption outside the home and frequency of food consumption in excess, from 1–7 (less than once in a month to all the days); anxiety (GAD-7 scale), from 0–3 (never to almost all the days); depression (CES-D scale), from 1–4 (none of the days to all the days); subscales from psychological well-being (PWB), from 1–6 (totally disagree to totally agree); emotional intelligence (TEIQUE scale), from 1–7 (totally disagree to totally agree), positive and negative emotions (PSS scale), from 1–5 (never to almost always) and optimism (LOT-R), from 1–5 (totally disagree to totally agree).

**Table 2:** Comparison between systolic blood pressure, diastolic blood pressure and anthropometric variables between the sexes.

Quantitatively Measured			
Variable, Mean ± SD	Women (n = 91)	Men (n = 80)	*p*-Value
Systolic blood pressure (SBP)	103.40 ± 12.11	121.96 ± 14.30	1.35 × 10^−16^
Diastolic blood pressure (DBP)	72.93 ± 7.6	78.61 ± 9.88	<0.001 *
Body mass index (BMI)	24.05 ± 3.72	24.80 ± 4.22	0.218
Waist-to-hip ratio (WHR)	0.77 ± 0.05	0.84 ± 0.07	<0.001 *
**Categorized BMI and WHR**			
**Body mass index (BMI)**			
Normal < 25 kg/m^2^	57 (62.6)	45 (56.2)	0.436
High > 25 kg/m^2^	34 (37.4)	35 (43.8)	
**Waist-to-hip ratio (WHR)**			
Normal: Women < 0.86 and Men < 0.90	86 (94.5)	64 (80.0)	0.005 *
High: Women ≥ 0.86 and Men ≥ 0.90	5 (5.5)	16 (20.0)	
**Categorized SBP and DBP**			
**Systolic blood pressure**			
Normal ≤ 120 mmHg	84 (92.3)	35 (43.7)	
Pre-hypertension (121–139 mmHg)	4 (4.4)	38 (47.5)	1.71 × 10^−12^
High ≥ 140 mmHg	3 (3.3)	7 (8.8)	
**Diastolic blood pressure**			
Normal ≤ 80 mmHg	76 (83.5)	47 (58.8)	
Pre-hypertension (81–89 mmHg)	13 (14.3)	24 (30.0)	0.002 *
High ≥ 90 mmHg	2 (2.2)	9 (11.2)	

* Significant values; *p*-values obtained with Student’s *t*-test and chi-squared test.

**Table 3:** Descriptive data of the laboratory variables studied and their comparison between the sexes.

Variable	Women (n = 91)	Men (n = 80)	Laboratory Reference Values	Participants Out of Range n (%)
Leukocytes (10^3^/μL), mean ± SD	6.80 ± 1.48	6.58 ± 1.81	5.00–10.00	28 (16.4)
- Lymphocytes	2.23 ± 0.53	2.17 ± 0.56	1.00–4.20	1 (0.6)
- Monocytes	0.49 ± 0.14	0.53 ± 0.16	0.10–1.00	1 (0.6)
- Neutrophils	3.88 ± 1.18	3.78 ± 1.44	1.50–7.00	4 (2.3)
- Eosinophils	0.14 ± 0.12	0.13 ± 0.09	0.05–0.40	20 (11.7)
- Basophils	0.04 ± 0.02	0.05 ± 0.02	0.01–0.05	42 (24.6)
Hemoglobin (g/dL), mean ± SD	13.79 ± 1.18	16.32 ± 0.79	W: 12.00–16.00, M: 14.00–17.00	W: 7 (7.7), M: 17 (21.3)
Hematocrit (%)	42.29 ± 3.19	48.48 ± 2.31	W: 36.0–48.0, M: 36.0–52.0	W: 9 (9.9), M: 5 (6.3)
Erythrocytes	4.71 ± 0.35	5.44 ± 0.33	W: 4.0–5.0, M: 4.5–6.2	W: 20 (22.0), M: 0 (0.0)
Platelets (10^3^/μL), mean ± SD	278.00 ± 53.61	260.18 ± 51.29	141.00–400.00	3 (1.8)
Glucose (g/dL), mean ± SD	87.30 ± 8.59	90.99 ± 14.81	74.00–106.00	11 (6.4)
Urea (mg/dL), mean ± SD	25.56 ± 6.52	28.44 ± 7.06	16.60–48.50	6 (3.5)
Blood urea nitrogen (BUN), mg/dL, mean ± SD	11.95 ± 3.04	13.29 ± 3.30	6.00–20.0	8 (4.6)
Creatinine (mg/dL), mean ± SD	0.74 ± 0.11	0.95 ± 0.13	W: 0.50–0.90	8 (8.8)
			M: 0.70–1.20	4 (5.0)
Uric acid (mg/dL), mean ± SD	4.21 ± 1.00	6.05 ± 1.10	W: 2.40–5.70	7 (7.7)
			M: 3.40–7.00	14 (17.5)
Lipid levels (mg/dL), mean ± SD				
Total cholesterol	166.18 ± 29.18	176.71 ± 36.43	≤200.00	36 (21.10)
High-density lipoprotein (HDL)	51.97 ± 11.72	44.47 ± 10.25	W ≥ 45.00, M ≥ 35.00	31 (34.1), 13 (16.3)
Low-density lipoprotein (LDL)	95.72 ± 24.19	108.96 ± 29.70	≤100.00	82 (48.0)
Triglycerides	92.59 ± 43.84	130.58 ± 97.97	≤150.00	34 (19.9)
Total proteins (g/dL)	7.47 ± 0.38	7.65 ± 0.39	6.4–8.3	5 (2.9)
Albumin (g/dL)	4.71 ± 0.26	5.01 ± 0.27	3.97–4.94	63 (36.8)
Liver enzymes (U/L), mean ± SD				
- AST	19.99 ± 16.10	29.93 ± 39.77	W ≤ 32.00, M ≤ 40.00	4 (4.4), 7 (8.6)
- ALT	17.52 ± 15.45	28.91 ± 21.20	W ≤ 33.00, M ≤ 41.00	6 (6.5), 11 (13.8)
- GGT	16.15 ± 7.62	27.58 ± 22.56	W ≤ 40.00, M ≤ 60.00	2 (2.2), 4 (5.0)
- ALP	78.61 ± 17.59	97.46 ± 26.99	W ≤ 104.00, M ≤ 129.00	5 (5.5), 12 (15.0)
- LDH	169.56 ± 32.03	187.03 ±100.98	W ≤ 214.00, M ≤ 225.00	4 (4.4), 5 (6.3)
Serum electrolytes				
- Calcium, mg/dL	9.71 ± 0.31	9.97 ± 0.34	8.6–10.0	39 (22.8)
- Phosphorus, mg/dL	3.62 ± 0.50	3.59 ± 0.50	2.5–4.5	6 (3.5)
- Magnesium, mg/dL	2.05 ± 0.11	2.08 ± 0.14	W: 1.7–2.2, M: 1.6–2.6	W: 2 (2.2), M: 0 (0.0)
- Iron, μg/dL	83.53 ± 36.81	116.58 ± 38.76	33.0–193.0	10 (5.8)
- Sodium, meq/L	139.05 ± 1.93	139.60 ± 1.97	136.0–145.0	5 (2.9)
- Potassium, meq/L	4.52 ± 0.41	4.45 ± 0.45	3.5–5.10	11 (6.4)
- Chloride, meq/L	103.78 ± 1.76	102.72 ± 1.91	98.0–107.0	4 (2.3)
Pancreatic enzymes				
Amilase, U/L	71.13 ± 25.65	72.24 ± 50.33	28.0–100.0	20 (11.7)
Lipase, U/L	34.52 ± 11.77	32.04 ± 20.12	13.0–60.0	4 (2.3)

AST: aspartate aminotransferase; ALT: alanine aminotransferase; GGT: gamma-glutamyl transferase; ALP: Alkaline phosphatase; LDH: lactate dehydrogenase; W: women; M: men.

**Table 4:** Significant bivariate correlations between the studied variables and systolic blood pressure and diastolic blood pressure in the global sample.

Variable	SBP (n =171)	DBP (n = 171)
Sex (Female = 1, male = 2)	0.607 **	0.322 **
With children	0.114	0.151 *
Erythrocytes	0.445 **	0.325 **
Hemoglobin	0.424 **	0.346 **
Hematocrit	0.400 **	0.336 **
Glucose	0.248 **	0.262 **
Creatinine	0.459 **	0.238 **
Uric acid	0.522 **	0.347 **
Cholesterol	0.208 **	0.123
Triglycerides	0.229 **	0.219 **
High-density lipoprotein (HDL)	−0.296 **	−0.231 **
Low-density lipoprotein (LDL)	0.275 **	0.175 *
Aspartate aminotransferase (AST)	0.241 **	0.128
Alanine aminotransferase (ALT)	0.288 **	0.215 **
Gamma-glutamyl transferase (GGT)	0.396 **	0.326 **
Alkaline Phosphatase (ALP)	0.304 **	0.178 *
Lactate Dehydrogenase (LDH)	0.193 *	0.135
Total proteins	0.282 **	0.250 **
Albumin	0.294 **	0.185 *
Calcium	0.222 **	0.171 *
Phosphorus	−0.246 **	−0.196 *
Magnesium	0.104	0.228 **
Lipase	−0.169 *	−0.033
Iron	0.158 *	0.102
Sodium	0.123	0.169 *
Chloride	−0.217 **	−0.113
Depression	−0.164 *	−0.104
Autonomy	0.221 **	0.162 *
Body mass index (BMI)	0.340 **	0.289 **
Waist/hip ratio (WHR)	0.482 **	0.341 **
Sleep satisfaction	0.197 **	0.120
Sleep quality	0.174 *	0.163 *

*p*-values obtained with Pearson and Spearman correlation tests. * *p*-value < 0.05; ** *p*-value < 0.01.

**Table 5:** Multivariate regression analysis for systolic blood pressure in the global sample.

Variable	B	Beta Coefficient	Significance	Change in R^2^	Tolerance
Constant	22.058	-	0.341		-
Male sex	8.870	0.276	0.001	0.334	0.380
Body mass index (BMI)	0.939	0.232	0.000	0.082	0.721
Age	0.347	0.230	0.000	0.026	0.621
Schooling	−4.837	−0.219	0.000	0.033	0.849
Albumin	13.731	0.258	0.000	0.030	0.575
Sleep satisfaction	0.981	0.091	0.085	0.015	0.932
Phosphorus	−5.230	-0.162	0.007	0.012	0.728
Monocytes	15.992	0.149	0.007	0.012	0.866
Eosinophils	−23.278	−0.156	0.005	0.014	0.868
Potassium	−5.483	−0.146	0.006	0.011	0.920
Glucose	0.186	0.139	0.014	0.013	0.824
Creatinine	4.978	0.146	0.048	0.010	0.479

R of the model: 0.770.

**Table 6:** Multivariate regression analysis for systolic blood pressure in women.

Variable	B	Beta Coefficient	Significance	Change in R^2^	Tolerance
Constant	67.277	-	0.000	-	-
Body mass index (BMI)	1.015	0.312	0.002	0.129	0.676
Age	0.697	0.604	0.000	0.055	0.282
Schooling	−5.623	−0.341	0.001	0.056	0.743
Monocytes	22.109	0.252	0.005	0.047	0.889
Eosinophils	−24.884	−0.252	0.005	0.049	0.871
Alkaline phosphatase (ALP)	−0.162	−0.235	0.011	0.027	0.840
Glucose	0.318	0.225	0.013	0.034	0.858
Emotion perception	−1.751	−0.209	0.026	0.025	0.794
With children	−8.330	−0.305	0.038	0.030	0.324

R of the model = 0.673.

**Table 7:** Multivariate regression analysis for systolic blood pressure in men.

Variable	B	Beta Coefficient	Significance	Change in R^2^	Tolerance
Constant	113.988	-	0.004	-	-
Schooling	−8.540	−0.433	0.000	0.062	0.654
Total proteins	10.486	0.284	0.005	0.125	0.665
Daily physical activity hours	6.894	0.434	0.000	0.076	0.828
Phosphorus	−16.906	−0.590	0.000	0.046	0.733
Potassium	−11.893	−0.375	0.000	0.048	0.813
Autonomy	6.228	0.426	0.000	0.038	0.652
Iron	−0.111	−0.300	0.001	0.031	0.817
Socioeconomic level	−11.160	−0.432	0.000	0.029	0.544
Monthly free money	3.256	0.302	0.008	0.024	0.524
Frequency of alcohol consumption	−3.257	−0.214	0.018	0.034	0.816
Calcium	8.317	0.196	0.054	0.026	0.639
Emotion perception	−1.862	−0.180	0.071	0.020	0.665

R of the model = 0.757.

**Table 8:** Multivariate regression analysis for diastolic blood pressure in the global sample.

Variable	B	Beta Coefficient	Significance	Change in R^2^	Tolerance
Constant	−13.916	-	0.737	-	-
Age	0.163	0.190	0.008	0.140	0.736
Hemoglobin	0.956	0.169	0.026	0.054	0.650
Glucose	0.198	0.259	0.000	0.046	0.835
Lactate dehydrogenase (LDH)	0.016	0.126	0.044	0.026	0.951
Blood urea nitrogen	−0.476	−0.168	0.009	0.026	0.921
Schooling	−2.589	−0.205	0.002	0.021	0.866
Body mass index (BMI)	0.450	0.195	0.004	0.015	0.814
Sodium	1.594	0.342	0.000	0.016	0.493
Chloride	−1.798	−0.373	0.000	0.017	0.462
Frequency of smoking consumption	−1.705	−0.137	0.030	0.013	0.929
Magnesium	8.862	0.122	0.069	0.025	0.836

R of the model: 0.644.

**Table 9:** Multivariate regression analysis for diastolic blood pressure in women.

Variable	B	Beta Coefficient	Significance	Change in R^2^	Tolerance
Constant	137.064	-	0.001	-	-
Uric acid	2.304	0.303	0.001	0.070	0.768
Lactate dehydrogenase (LDH)	0.066	0.276	0.001	0.083	0.887
Phosphorus	−3.916	−0.256	0.007	0.048	0.658
With partner	−3.910	−0.258	0.005	0.042	0.689
Daily physical activity hours	−2.783	−0.337	0.000	0.043	0.780
Hemoglobin	1.839	0.286	0.001	0.041	0.808
Personal growth	−1.796	−0.228	0.010	0.036	0.749
Age	0.452	0.622	0.000	0.032	0.257
Daily free hours	0.806	0.235	0.007	0.045	0.787
Sleep satisfaction	0.874	0.166	0.053	0.026	0.776
Triglycerides	−0.051	−0.293	0.003	0.025	0.590
Body mass index (BMI)	0.560	0.274	0.008	0.019	0.545
Waist-to-hip ratio (WHR)	−27.736	−0.175	0.078	0.022	0.575
Chloride	−0.840	−0.194	0.017	0.022	0.875
With children	−4.526	−0.263	0.051	0.021	0.313
Frequency of smoking consumption	−1.589	−0.138	0.092	0.016	0.854

R of the model: 0.768.

**Table 10:** Multivariate regression analysis for diastolic blood pressure in men.

Variable	B	Beta Coefficient	Significance	Change in R^2^	Tolerance
Constant	−1.733	-	0.937	-	-
With children	10.350	0.363	0.001	0.135	0.848
Total proteins	6.194	0.242	0.021	0.064	0.846
Quality of food intake	−1.011	−0.244	0.013	0.042	0.965
Magnesium	14.325	0.202	0.057	0.046	0.818
Self-motivation	1.538	0.187	0.055	0.033	0.977
Aspartate aminotransferase (AST)	0.045	0.181	0.072	0.030	0.907

R of the model: 0.591.

## Data Availability

The raw data presented in this study are available on request to the corresponding author. The data are not publicly available due to the database has a specific codification that needs to be explained by the researcher responsible of the study. In addition, all sensible data of the participants in the database must be eliminated from it in order to be send to another researcher.

## Supplementary Materials

The following supporting information can be downloaded at: https://www.mdpi.com/article/10.3390/jcm13020378/s1.

## References

[1] Galeano I.O., Franquelo-Morales P., Notario-Pacheco B., Rodríguez J.A.N., Cañete M.U., Martínez-Vizcaíno V. (2012). Prehipertensión arterial en adultos jóvenes. Rev. Clin. Esp..

[2] Trudel-Fitzgerald C., Gilsanz P., Mittleman M.A., Kubzansky L.D. (2015). Dysregulated Blood Pressure: Can Regulating Emotions Help?. Curr. Hypertens. Rep..

[3] Filippini T., Naska A., Kasdagli M., Torres D., Lopes C., Carvalho C., Moreira P., Malavolti M., Orsini N., Whelton P.K. (2020). Potassium intake and bood pressure: A Dose-Response Meta-Analysis of Randomized Controlled Trials. J. Am. Heart Assoc..

[4] Yanes L.L., Romero D.G., Iliescu R., Zhang H., Davis D., Reckelhoff J.F. (2010). Postmenopausal hypertension: Role of the renin-angiotensin system. Hypertension.

[5] Casilimas G.A.G., Martin D., Martínez M.A., Merchán C.R., Mayorga C., Barragán A.F. (2017). Fisiopatología de la hipertensión arterial secundaria a obesidad. Arch. Cardiol. México.

[6] McClure S.T., Rebholz C.M., Mitchell D.C., Selvin E., Appel L.J. (2019). The association of dietary phosphorus with blood pressure: Results from a secondary analysis of the PREMIER trial. J. Hum. Hypertens..

[7] Scalco A.Z., Scalco M.Z., Azul J.B.S., Neto F.B. (2005). Hypertension and depression. Clinics.

[8] Brugnera A., Compare A., Omboni S., Greco A., Carrara S., Tasca G., Poletti B., Parati G. (2022). Psychological covariates of blood pressure among patients with hypertension and metabolic syndrome. Health Psychol..

[9] Shukla M., Pandey R., Jain D., Lau J.Y.F. (2017). Poor emotional responsiveness in clinical hypertension: Reduced accuracy in the labelling and matching of emotional faces amongst individuals with hypertension and prehypertension. Psychol. Health.

[10] Handan D., Hakan D., Puşuroğlu M., Seyda Yılmaz A. (2022). Anxiety disorders and depression are associated with resistant hypertension. Adv. Clin. Exp. Med..

[11] Auer A., von Känel R., Lang L.L., Thomas L., Zuccarella-Hackl C., Degroote C., Gideon A., Wiest R., Wirtz P.H. (2022). Do Hypertensive Men Spy with an Angry Little Eye? Anger Recognition in Men with Essential Hypertension—Cross-sectional and Prospective Findings. Ann. Behav. Mes..

[12] Baker L.A.A., Aldin S.Z.J. (2022). Association of some biochemical parameters and blood pressure among males with hypertension in the camps of Nineveh province-Iraq. J. Popul. Ther. Clin. Pharmacol..

[13] Baldo M.P., Brant L.C.C., Cunha R.S., Del Carmen Bisi Molina M., Griep R.H., Barreto S.M., Lotufo P.A., Bensenor I.M., Mill J.G. (2019). The association between salt intake and arterial stiffness is influenced by a sex-specific mediating effect through blood pressure in normotensive adults: The ELSA-Brasil study. J. Clin. Hypertens..

[14] Díaz-Pértegas S., Pita-Fernández S. (2022). Determinación del tamaño muestral para calcular la significación del coeficiente de correlación lineal. Cad. Aten. Primaria.

[15] Bobes-García J., González G., Portilla P., Sáiz-Martínez D.A., Bascarán-Fdez M., Iglesias-Álvarez G., Fdez-Domínguez J.M. (2000). Propiedades psicométricas del cuestionario de Oviedo de Sueño. Psicothema.

[16] Bernal-Orozco M., Badillo-Camacho N., Macedo-Ojeda G., González-Gómez M., Orozco-Gutiérrez J., Prado-Arriaga R., Márquez-Sandoval F., Altamirano-Martínez M., Vizmanos B. (2018). Design and Reproducibility of a Mini-Survey to Evaluate the Quality of Food Intake (Mini-ECCA) in a Mexican Population. Nutrients.

[17] Márquez-Sandoval Y.F., Salazar-Ruiz E.N., Macedo-Ojeda G., Altamirano-Martínez M.B., Bernal-Orozco M.F., Salas-Salvadó J., Vizmanos B. (2014). Design and validation of a questionnaire to assess dietary behavior in Mexican students in the area of health. Nutr. Hosp..

[18] Radloff L. (1977). The CES-D Scale: A self-report depression scale for research in the general population. Appl. Psychol. Meas..

[19] Bojorquez-Chapela L., Salgado de Snyder N. (2009). Características psicométricas de la escala Center for Epidemiological Studies-Depression (CES-D), versiones de 20 y 10 reactivos en mujeres de una zona rural mexicana. Salud Ment..

[20] Garcia-Campayo J., Zamorano E., Ruiz M.A., Pardo A., Perez-Paramo M., Lopez-Gomez V., Freire O., Rejas J. (2010). Cultural adaptation into Spanish of the generalized anxiety disorder-7 (GAD-7) scale as a screening tool. Health Qual. Life Outcomes.

[21] Cortina-Guzmán L.G., Berenzon-Gom S. (2013). Traducción al español y propiedades picométricas del instrumento “positivity self test”. Psicol. Iberoam..

[22] Diaz D., Rodriguez-Carvajal R., Blanco A., Moreno-Jimenez B., Gallardo I., Valle C., Van Dierendonck D. (2006). Adaptación española de las escalas de bienestar psicológico de Ryff. Psicothema.

[23] Ferrando P.J., Chico E., Tous J.M. (2002). Propiedades psicométricas del test de Optimismo Life Orientation Test. Psicothema.

[24] Chirumbolo A., Picconi L., Morelli M., Petrides K.V. (2019). The Assessment of Trait Emotional intelligence: Psychometric Characteristics of the TEIQue-Full Form in a Large Italian Adult Sample. Front. Psychol..

[25] Chobanian A.V., Bakris G.L., Black H.R., Cushman W.C., Green L.A., Izzo J.L. (2003). The seventh report of the Joint National Committee on prevention, detection, evaluation, and treatment of high blood pressure: The JNC 7 report. J. Am. Med. Assoc..

[26] Lear S.A., James P.T., Ko G.T., Kumanyika S.K. (2010). Appropriateness of waist circumference and waist-to-hip ratio cutoffs for different ethnic groups. Eur. J. Clin. Nutr..

[27] Dong G., Wang D.H., Liu M., Liu Y., Zhao Y., Yang M., Meng X., Tian S., Meng X., Zhang H. (2012). Sex difference of the prevalence and risk factors associated with prehypertension among urban Chinese adults from 33 communities of China. J. Hypertens..

[28] Kringeland E., Tell G.S., Midtbo H., Igland J., Haugsgjerd T.R., Gerdts E. (2022). Stage 1 hypertension, sex, and acute coronary syndromes during midlife: The Hordaland health study. Eur. J. Prev. Cardiol..

[29] Ji H., Niiranen T.J., Rader F., Henglin M., Kim A., Ebinger J.E., Claggett B., Merz C.N.B., Cheng S. (2021). Sex differences in blood pressure associations with cardiovascular outcomes. Circulation.

[30] Nwankwo T., Yoon S.S., Burt V., Gu Q. (2013). Hypertension among Adults in the United States: National Health and Nutrition Examination Survey, 2011–2012.

[31] Joyner M.J., Wallin B.G., Charkoudian N. (2015). Sex differences and blood pressure regulation in humans. Exp. Physiol..

[32] Molina L., Elosúa R., Marrugat J., Pons S. (1999). Relation of maximum blood pressure during exercise and regular physical activity in normotensive men with left ventricular mass and hypertrophy. Am. J. Cardiol..

[33] (1997). The sixth report of the Joint National Committee on prevention, detection, evaluation, and treatment of high blood pressure. Arch. Intern. Med..

[34] Li C., Shang S. (2021). Relationship between Sleep and Hypertension: Findings from the NHANES (2007–2014). Int. J. Environ. Res. Public Health.

[35] Shan M.M., Mandiga P. (2022). Psyshiology, Plasma, Osmolality and Oncotic Pressure.

[36] Chan Q., Stamler J., Griep L.M.O., Daviglus M.L., Van Horn L., Elliott P. (2016). An update on nutrients and blood pressure. J. Atheroscler. Thromb..

[37] Behradmanesh S., Nasri H. (2013). Association of serum calcium with level of blood pressure in type 2 diabetic patients. J. Nephropathol..

[38] Farsana Y., Samad N. (2020). REPORT-Association between serum electrolytes and erythrocytes Na+, K+ in hypertensive and normotensive male compared to female. Pak. J. Pharmacol. Sci..

[39] Siedlinski M., Józefczuk E., Xu X., Teumer A., Evangelou E., Schnabel R.B., Welsh P., Maffia P., Erdmann J., Tomaszewski M. (2020). White blood cells and blood pressure. Circulation.

[40] Lee S., Rim J.H., Kim J. (2015). Association of hemoglobin levels with blood pressure and hypertension in a large population-based study: The Korea National Health and Nutrition Examination Surveys 2008–2011. Clin. Chim. Acta.

[41] De Bacquer D., De Backer G., De Buyzere M., Kornitzer M. (1998). Is low serum chloride level a risk factor for cardiovascular mortality?. J. Cardiovasc. Risk.

[42] Hatzistavri L., Sarafidis P.A., Georgianos P.I., Tziolas I.M., Aroditis C.P., Zebekakis P., Pikilidou M., Lasaridis A.N. (2009). Oral magnesium supplementation reduces ambulatory blood pressure in patients with mild hypertension. Am. J. Hypertens..

[43] Bawazier L.A., Sja’bani M., Irijanto F., Zulaela Z., Widiatmoko A., Kholiq A., Tomino Y. (2020). Association of serum uric acid, morning home blood pressure and cardiovascular risk factors in a population with previous prehypertension: A cross-sectional study. BMJ Open.

[44] Li G., Wang H., Wang K., Wang W., Dong F., Qian Y., Gong H., Hui C., Xu G., Li Y. (2017). The association between smoking and blood pressure in men: A cross-sectional study. BMC Public Health.

